# Chemopreventive effects of 5-aminosalicylic acid on inflammatory bowel disease-associated colorectal cancer and dysplasia: a systematic review with meta-analysis

**DOI:** 10.18632/oncotarget.13715

**Published:** 2016-11-30

**Authors:** Xinyun Qiu, Jingjing Ma, Kai Wang, Hongjie Zhang

**Affiliations:** ^1^ Department of Gastroenterology, First Affiliated Hospital of Nanjing Medical University, Nanjing, Jiangsu 210029, China; ^2^ Institute of Apicultural Research, Chinese Academy of Agricultural Sciences, Beijing 100093, China

**Keywords:** 5-Aminosalicylic acid, inflammatory bowel disease, chemopreventive effect, colorectal cancer, dysplasia

## Abstract

**Background and Aims:**

The chemopreventive effect of 5-aminosalicylic acid (5-ASA) in patients with inflammatory bowel disease (IBD) has been widely studied; however, the results remain conflicting. The aim of this study was to systematically review the literature and update evidence concerning effects of 5-ASA on the risk of colorectal cancer (CRC) and dysplasia (Dys) in patients with ulcerative colitis (UC) or Crohn's disease (CD).

**Results:**

5-ASA showed a chemopreventive effect against CRC/Dys in IBD patients (OR = 0.58, 95% CI: 0.45−0.75). However, this effect was significant only in clinical-based studies (OR = 0.51; 95% CI: 0.39−0.65), but not in population-based studies (OR = 0.71; 95% CI: 0.46−1.09). Moreover, this effect was noticeable in patients with UC (OR = 0.46, 95% CI: 0.34−0.61), but not in CD (OR = 0.66, 95% CI: 0.42−1.03), and on the outcome of CRC (OR = 0.54, 95% CI: 0.39−0.74), but not Dys (OR = 0.47; 95% CI: 0.20−1.10). In IBD patients, mesalazine dosage ≥ 1.2 g/day showed greater protective effects against CRC/Dys than dosages < 1.2 g/day. However, Sulphasalazine therapy did not show any noticeable protective function regardless of the dosage administered.

**Materials and Methods:**

We performed a systematic review with a meta-analysis of 26 observational studies involving 15,460 subjects to evaluate the risks of developing CRC and Dys in IBD patients receiving 5-ASA treatment. Pooled odds ratios (ORs) and 95% confidence intervals (CIs) were calculated for each evaluation index.

**Conclusions:**

5-ASA has a chemopreventive effect on CRC (but not Dys) in IBD patients. Moreover, UC patients can benefit more from 5-ASA than CD patients. Mesalazine maintenance dosage ≥ 1.2 g/day is an effective treatment for reducing CRC risk in IBD patients.

## INTRODUCTION

Inflammatory bowel disease (IBD) comprises two major phenotypes: Crohn's disease (CD) and ulcerative colitis (UC). IBD is usually associated with an increased risk of colorectal cancer (CRC) [1, 2]. Previous studies have found that the cumulative risk of CRC in patients with UC was 2% at 10 years, 8% at 20 years, and 18% at 30 years after the diagnosis [3], whereas the relative risk of developing CRC was 4.5-fold higher in patients with CD than in healthy subjects [4]. Increasing evidence suggests that chronic colonic inflammation is an important trigger for colon carcinogenesis [5]. Therefore, improving the intestinal inflammatory conditions could be an effective method for preventing the development of IBD and some relevant malignant diseases [6].

5-Aminosalicylic acid (5-ASA, the main metabolite of sulphasalazine, balsalazide, and olsalazine) is a well-tolerated anti-inflammatory drug that can prevent intestinal inflammation and induce mucosal healing. It is commonly prescribed for treating IBD, and previous studies have reported the antineoplastic activities of 5-ASA in the gut [5]. Pinczowski *et al.* (1994) reported the first clinical findings on the use of 5-ASA for preventing the progression of UC to CRC. The authors showed that sulphasalazine decreased the risk of CRC in patients with UC by 62% [7]. These findings have provoked great interest in researchers and led to conflicting findings on the chemopreventive effects of 5-ASA in IBD. Some studies reported that 5-ASA significantly decreased the morbidity associated with CRC in IBD patients [8–12], whereas other studies claimed that the drug had an insignificant protective role [13–17]. Previous studies on the effects of 5-ASA in patients with IBD were conducted using population-based or clinical-based data, either of which can introduce more selection bias into an analysis, especially the latter [18]. Furthermore, the chemopreventive effects of 5-ASA could be different in CRC and dysplasia (Dys) in patients with UC or CD, in addition, the dosage of 5-ASA varies among studies and geographical regions, therefore, these confounding factors should be analyzed separately. Five new studies comprising 2,655 subjects have been published in the past few years. [12, 19–22] To the best of our knowledge, these studies have not been included in any of the published meta-analyses when evaluating the effect of 5-ASA on the risks of developing CRC and Dys in IBD patients [18, 23–25].

In this study, we performed a systematic review with meta-analysis to evaluate the effect of 5-ASA on the risks of developing CRC and Dys (the precancerous lesions of CRC [14]), with the aims of further understanding the roles of 5-ASA in chemo-prevention of IBD-associated CRC and Dys in UC and CD. Moreover, the effect of high and low dosages of 5-ASA in preventing the development of malignant outcomes and chemopreventive effect of 5-ASA in different geographical regions were evaluated.

## RESULTS

### Literature search and characteristics of the included studies

**Figure 1:**
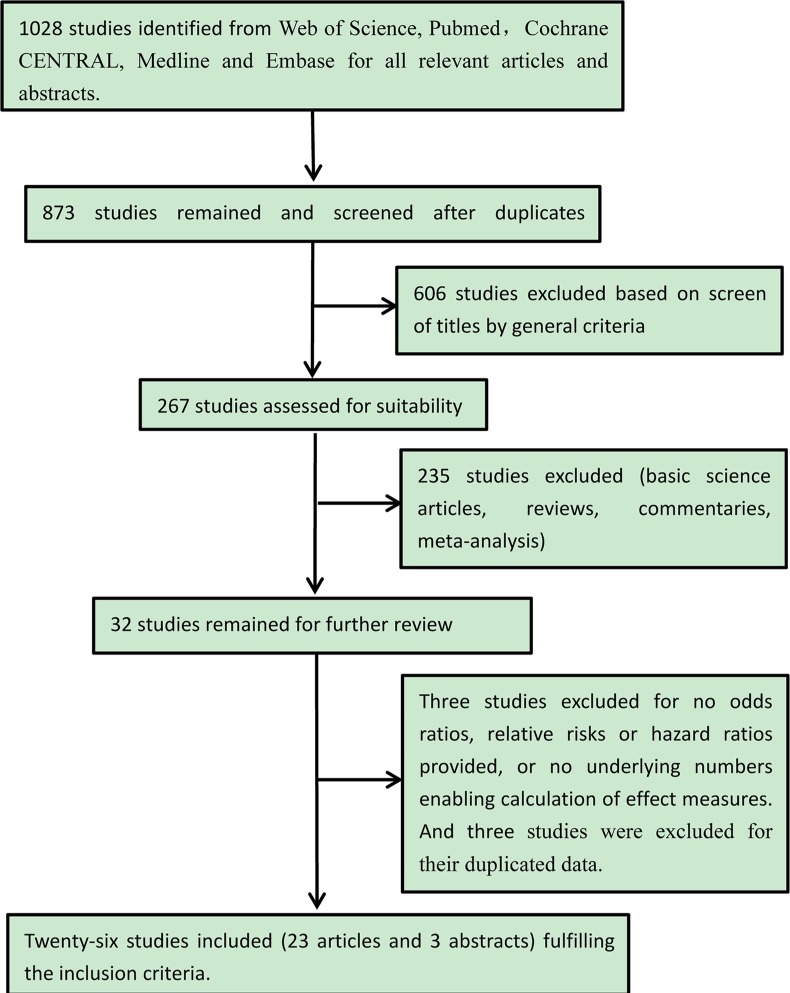
Flow chart of the literature search

The search results are summarised in Figure 1. We reviewed 1,028 titles and abstracts from the databases mentioned above. Duplicate records, basic science articles, reviews, commentaries, meta-analyses, and studies that did not provide ORs, RRs, or HRs, or the relevant data to enable the calculations of those effect measures were excluded. Twenty-nine papers were further assessed for suitability. Three studies were excluded as their data were duplicated [26–28]. In total, 23 full texts and 3 abstracts were reviewed. Of these, 15 studies were exclusively on UC patients; 1 was exclusively on CD patients, and 10 were on both UC and CD (UC/CD) patients. four [11, 15, 19, 22] of the 10 studies on the UC/CD patients reported ORs for both groups of patients separately. Altogether, there were 1,958 cases (IBD patients who developed CRC or Dys) and 13,492 controls (IBD patients who did not develop CRC or Dys) in all of the studies included in the primary analysis. The characteristics of each study are outlined in Table 1.

**Table 1:** Characteristics of the included studies

Study	Year	Study period	Cases /Controls	distribution of IBD diagnosis	Study design	Categories of 5-ASA	Outcome	Setting	Country	Newcastl-ottawa Score
Pinczowski, et al.	1994	1965–1983	102/196	UC	Case-control	SASP	CRC	Population-based	Sweden	7
Moody,et al.	1996	1972–1992	10/158	UC	Cohort	SASP	CRC	Population-based	UK	5
Lashner, et al.	1997	1986–1992	29/69	UC	Cohort	SASP/Mesa	CRC/Dys	Hospital-based	USA	6
Eaden, et al.	2000	1972–1989	102/102	UC	Case-control	SASP/Mesa	CRC	Hospital-based	UK	6
Lindberg, et al.	2001	1973–1993	50/92	UC	Cohort	SASP	CRC/Dys	Hospital-based	Sweden	4
Rutter, et al.	2004	1988–2002	68/136	UC	Case-control	SASP/non-SASP	CRC/Dys	Hospital-based	UK	6
van staa, et al.	2005	1987–2001	100/600	UC/CD	Case-control	SASP/Mesa/Balsa/Olsa	CRC	Population-based	UK	8
Smith, et al.^#^	2006	1995–2005	7/17	UC	Cohort	Mesa	Dys	Hospital-based	USA	4
Siegel, et al.	2006	1990–2004	27/27	CD	Case-control	5-ASA	CRC	Hospital-based	USA	6
Velayos, et al.	2006	1976–2002	188/188	UC	Case-control	SASP/Mesa/Balsa/Olsa	CRC	Hospital-based	USA	7
Gupta, et al.	2007	1996–2007	65/353	UC	Cohort	Mesa-based agents	CRC/Dys	Hospital-based	USA	5
Jess, et al.	2007	1940–2002	43/102	UC/CD	Case-control	SASP/Mesa	CRC/Dys	Population-based	USA+Denmark	8
Terdiman, et al.	2007	2000–2003	364/1172	UC/CD	Case-control	SASP/Mesa/Balsa	CRC	Population-based	USA	8
Ullman, et al.	2008	1996–1997	17/294	UC	Cohort	SASP/Mesa/Balsa/Olsa	CRC/Dys	Hospital-based	USA	5
Tang,et al.	2010	1970–2005	18/30	UC/CD	Case-control	Mesa	CRC	Hospital-based	USA	6
Carrat, et al.^#^	2010	2007–2007	153/304	UC/CD	Case-control	5-ASA	CRC	Hospital-based	France	5
Baars, et al.	2011	1990–2006	173/392	UC/CD	Case-control	5-ASA	CRC	Population-based	Netherlands	6
Bernstein, et al.	2011	1995–2008	101/303	UC/CD	Cohort	SASP/Mesa/Olsa	CRC	Population-based	Canada	8
Soon, et al.^#^	2011	Unknown	8/138	UC	Cohort	SASP/Mesa	CRC/Dys	Hospital-based	Singapore (Chinese,India,Malays)	6
Gong, et al.	2012	1998–2009	34/3888	UC	Case-control	SASP/Mesa	CRC	Hospital-based	China	5
van Schaik, et al.	2012	2001–2009	28/2578	UC/CD	Cohort	5-ASA	Dys	Population-based	Netherlands	8
Rubin, et al.	2013	1994–2005	59/141	UC	Case-control	SASP/Mesa	CRC/Dys	Hospital-based	USA	6
Nieminen,et al.	2014	1996–2008	183/370	UC/CD	Case-control	5-ASA	CRC/Dys	Hospital-based	Finland	7
Zhang,et al.	2015	2000–2012	4/620	UC	Cohort	5-ASA	CRC	Hospital-based	China	5
Nowacki,et al.	2015	2002–2013	10/424	UC	Cohort	Mesa	CRC	Hospital-based	Germany	5
Cheddani, et al.	2016	1988–2006	15/829	UC/CD	Case-control	5-ASA	CRC	Population-based	France	6

UC, ulcerative colitis; CD, Crohn's disease; SASP: sulfasalazine; Mesa: mesalazine; Balsa: balsalazide; Olsa: Olsalazine; CRC, colorectal cancer; Dys, dysplasia.

# studies reported in abstract form.

### Effect of 5-ASA treatment on the risk of CRC/Dys in patients with IBD (UC/CD)

**Figure 2:**
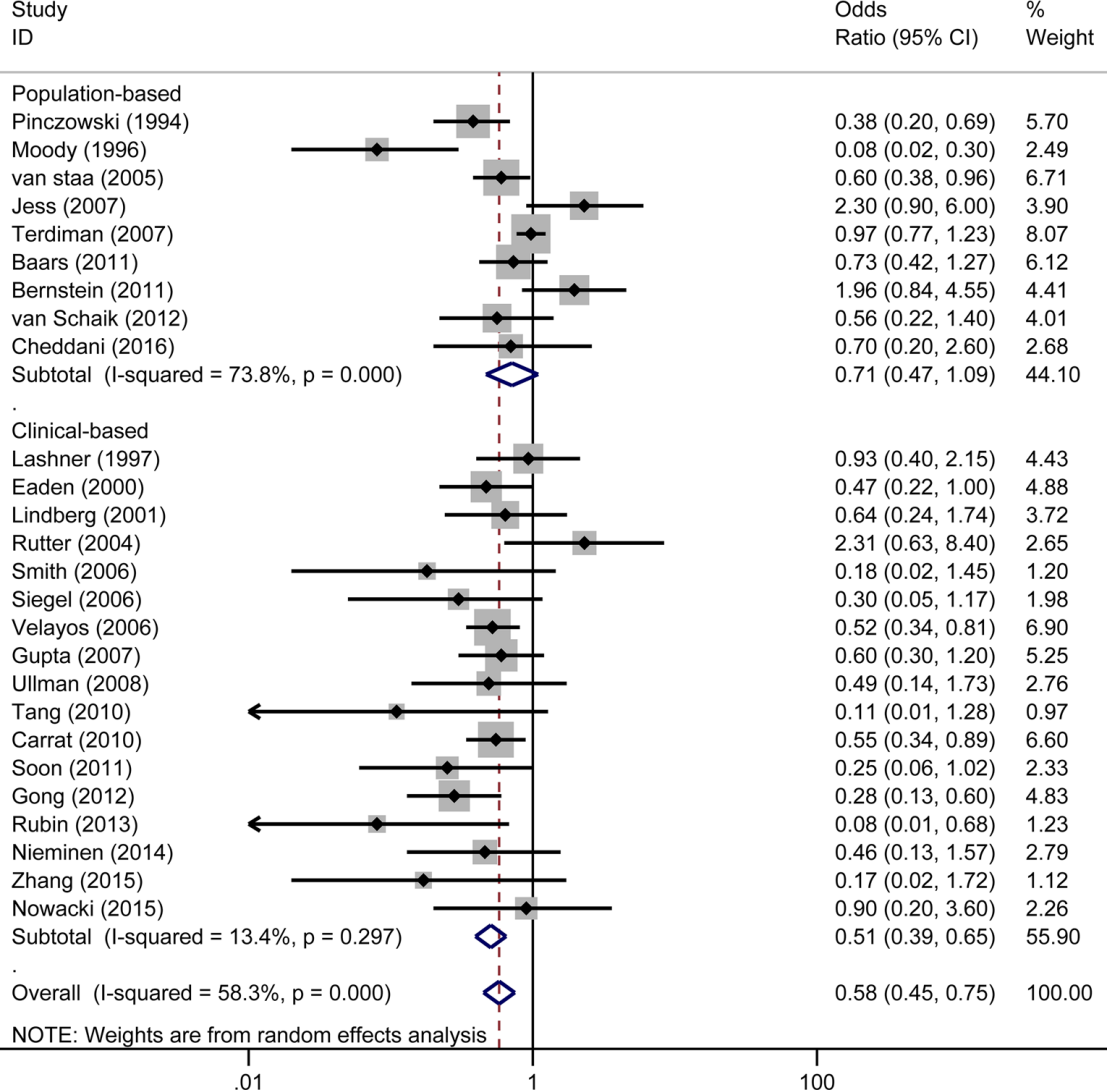
Forest plot of sub-analyses of 5-ASA on the risk of CRC/Dys in patients with IBD (UC/CD) between population-based and clinical-based cohort studies Size of boxes is proportional to weight of the individual study. The relative weighted contribution of each of the studies to the overall pooled adjusted odds ratio is shown on the far right. CI, confidence interval.

**Figure 3:**
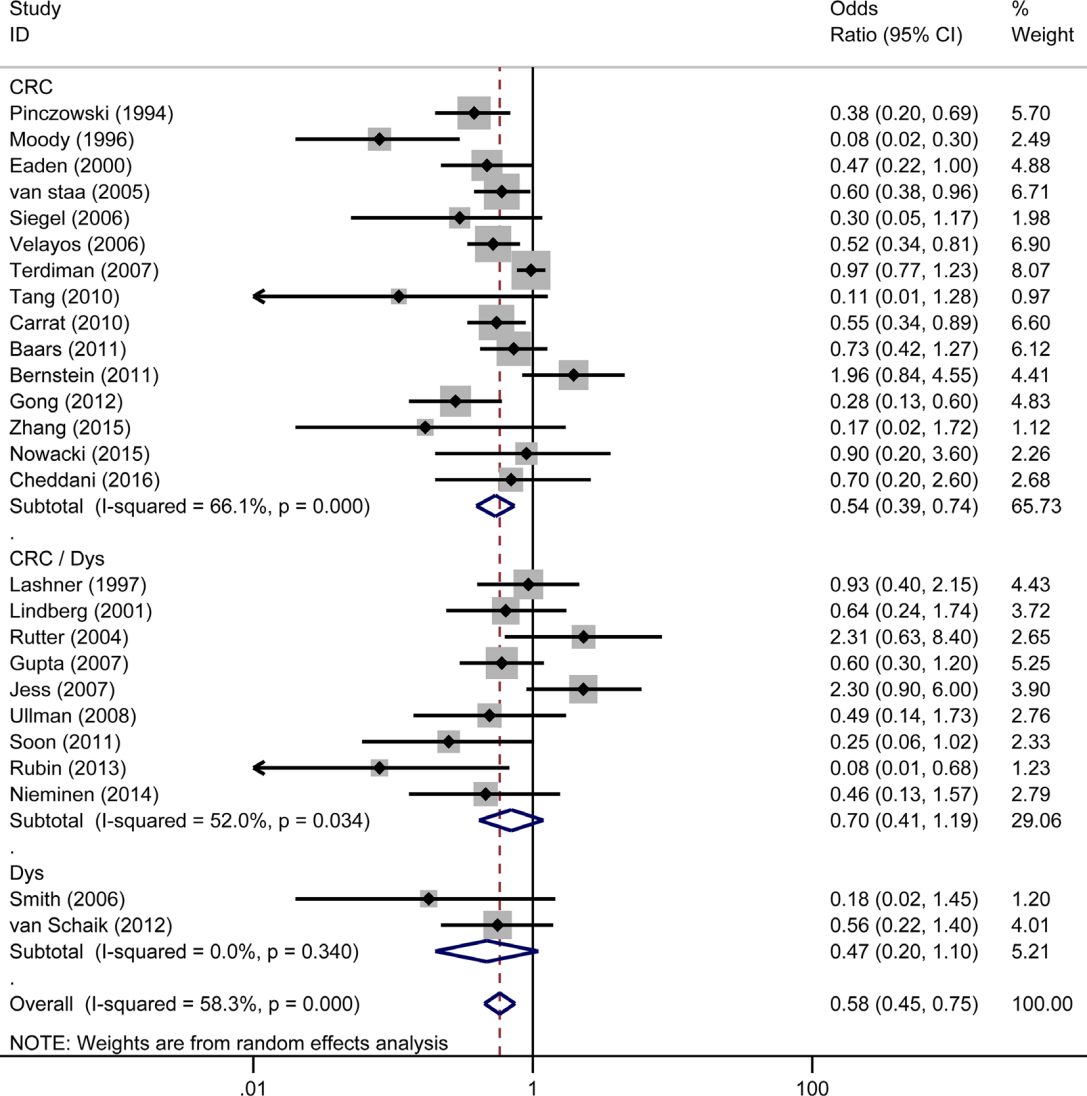
Forest plot of sub-analyses of 5-ASA on the risk of CRC, Dys and CRC/Dys in patients with IBD (UC/CD)

Nine studies [7–9, 14, 15, 17, 22, 29, 30] were regarded as population-based (or large administrative database samples) and 17 studies [10–13, 19–21, 31–40] as clinical-based. In a pooled analysis of all of the 26 studies, 5-ASA was associated with a decreased risk of CRC/Dys (OR = 0.58, 95% CI: 0.45–0.75, *I^2^* = 58.3%, *P* = 0.000) (Figure 2). While divided total studies into two subgroups by different settings, 5-ASA use was associated with a reduced risk of CRC/Dys in the clinical-based (OR_clinical-based_ = 0.51, 95% CI: 0.39–0.65, *I^2^* = 13.4%, *P* = 0.297) studies but not in the population-based studies (OR_population-based_ = 0.71, 95% CI: 0.46–1.09, *I^2^* = 73.8%, *P* = 0.000) (Figure 2). Dys and CRC are different pathological stages in the development of colonic carcinoma. Some studies [7–11, 15–17, 20–22, 32, 34, 38, 40] reported only the outcome of CRC, some [35, 41] reported only the outcome of Dys, while others [12–14, 19, 31, 33, 36, 37, 39] reported the combined outcome (CRC/Dys). In this meta-analysis, we further divided the studies into three subgroups according to the different reported outcomes (CRC, Dys, and CRC/Dys). 5-ASA significantly decreased the risk of CRC (OR_CRC_ = 0.54, 95% CI: 0.39–0.74, *I^2^* = 66.1%, *P* = 0.000); however, it did not reduce the risk of Dys or CRC/Dys in patients with IBD (OR_Dys_ = 0.47, 95% CI: 0.20–1.10, *I^2^* = 0.0%, *P* = 0.34; OR_CRC/Dys_ = 0.70, 95% CI: 0.41–1.19, *I^2^* = 52.0%, *P* = 0.034) (Figure 3).

### Effect of 5-ASA treatment on the risk of CRC/Dys in patients with UC

**Figure 4:**
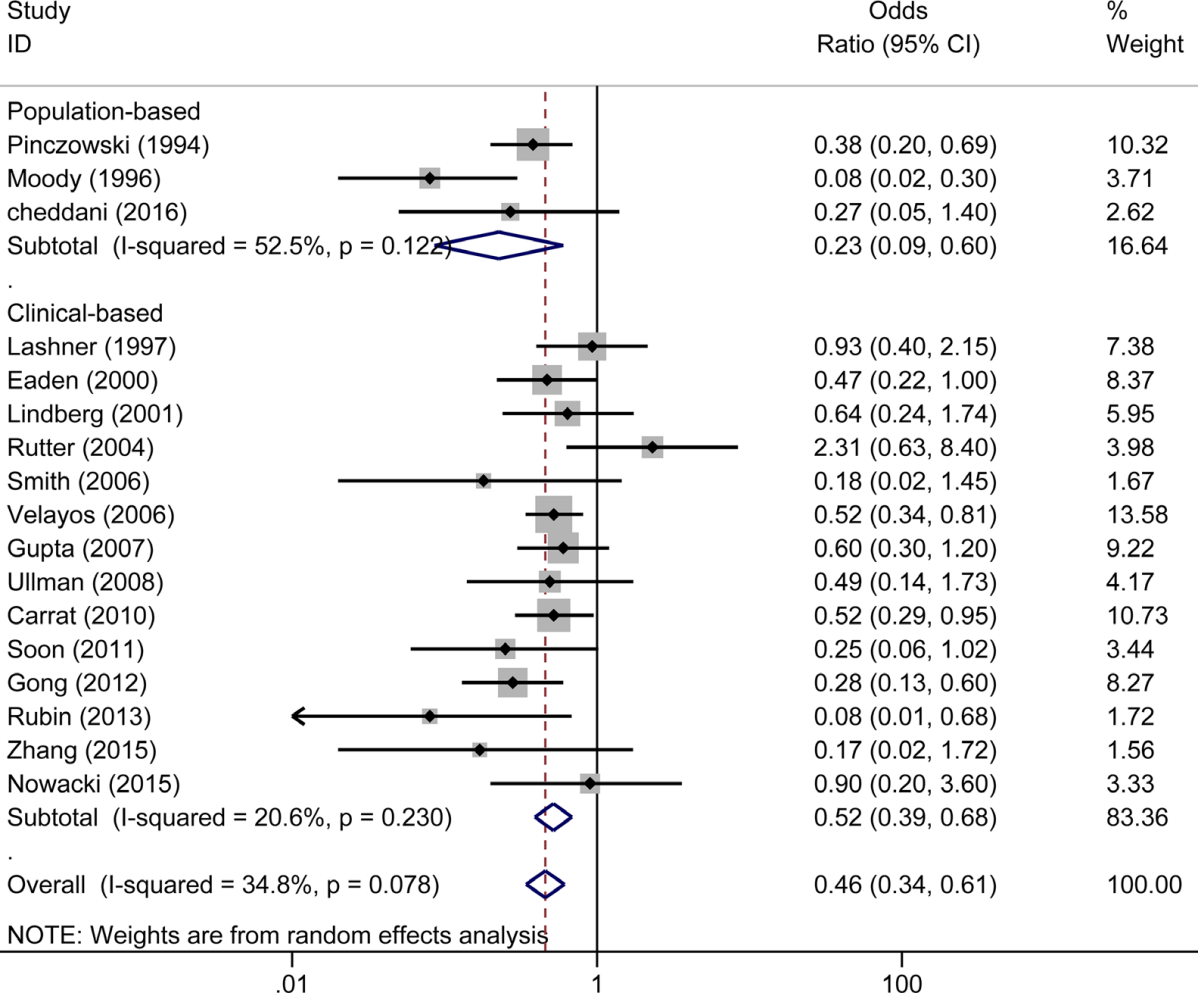
Forest plot of sub-analyses of 5-ASA on the risk of CRC/Dys in patients with UC between population-based and clinical-based cohort studies

**Figure 5:**
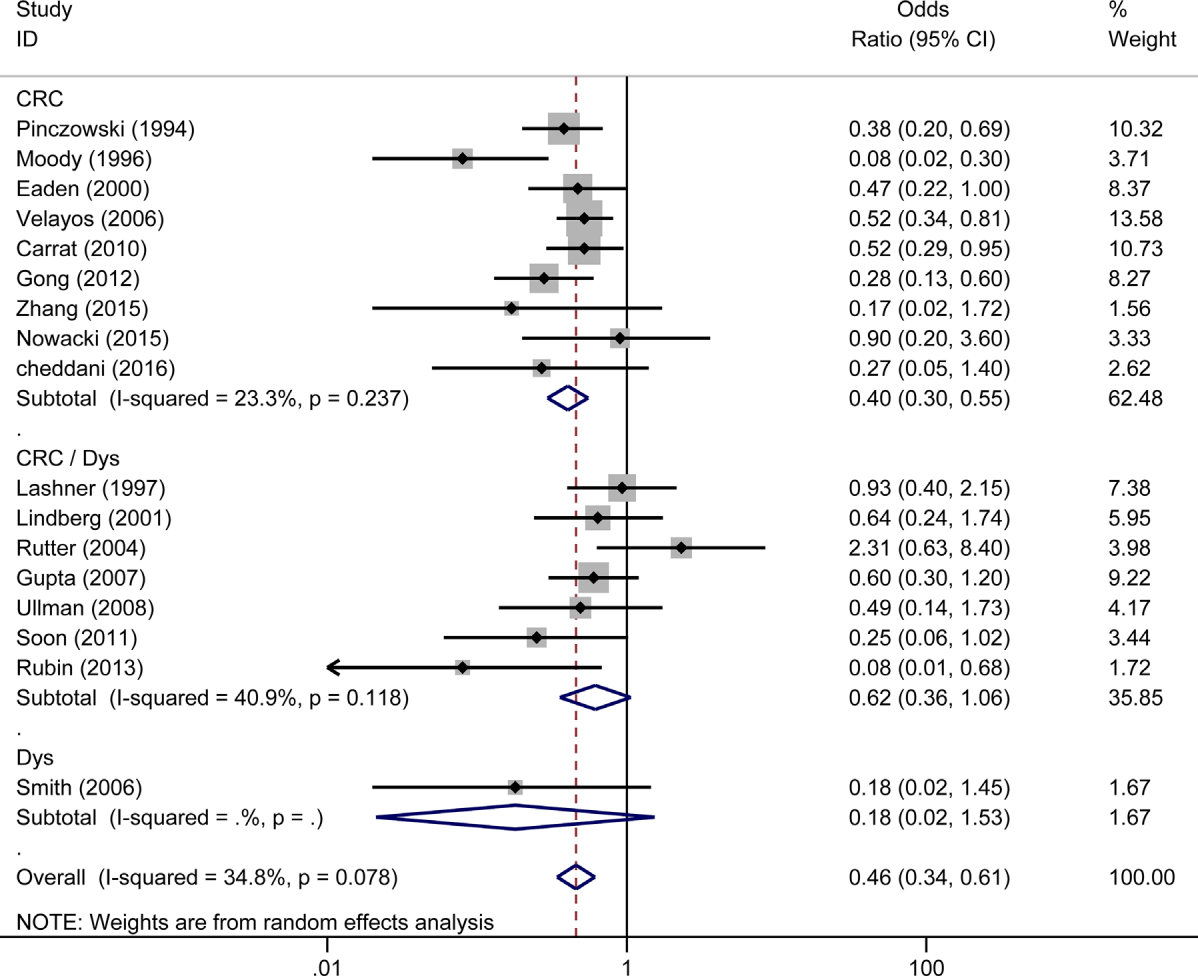
Forest plot of sub-analyses of 5-ASA on the risk of CRC, Dys and CRC/Dys in patients with UC

Seventeen studies reported the effect of 5-ASA on the risk of CRC/Dys in patients with UC. Three [7, 8, 22] of the 17 studies were population-based studies and showed that 5-ASA exhibited protective effects against the risk of CRC/Dys (OR _population-based_ = 0.23, 95% CI: 0.09–0.60, *I^2^* = 52.5%, *P* = 0.122) (Figure 4). The remaining fourteen clinical-based studies [10–13, 20, 21, 31–33, 35–37, 39, 40] also showed a pooled beneficial effect of 5-ASA on the risk of CRC/Dys (OR_clinical-based_ = 0.52, 95% CI: 0.39–0.68, *I^2^* = 20.6%, *P* = 0.230) (Figure 4). Nine of the 17 studies used CRC as an outcome [7, 8, 10, 11, 20–22, 32, 40], revealing a protective effect of 5-ASA on CRC risk (OR_CRC_ = 0.40, 95% CI: 0.30–0.55, *I^2^* = 23.3%, *P* = 0.237). Only one study reported Dys as an outcome [35] did not show a significant preventive function of 5-ASA (OR_Dys_ = 0.18, 95% CI: 0.02–1.53). Seven studies used Dys and CRC as a combined outcome [12, 13, 31, 33, 36, 37, 39], yielding a pooled OR of 0.62 (95% CI: 0.36–1.06, *I^2^* = 40.9%, *P* = 0.118) which was not indicative of a significant protective effect of 5-ASA (Figure 5).

### Effect of 5-ASA treatment on the risk of CRC/Dys in patients with CD

**Figure 6:**
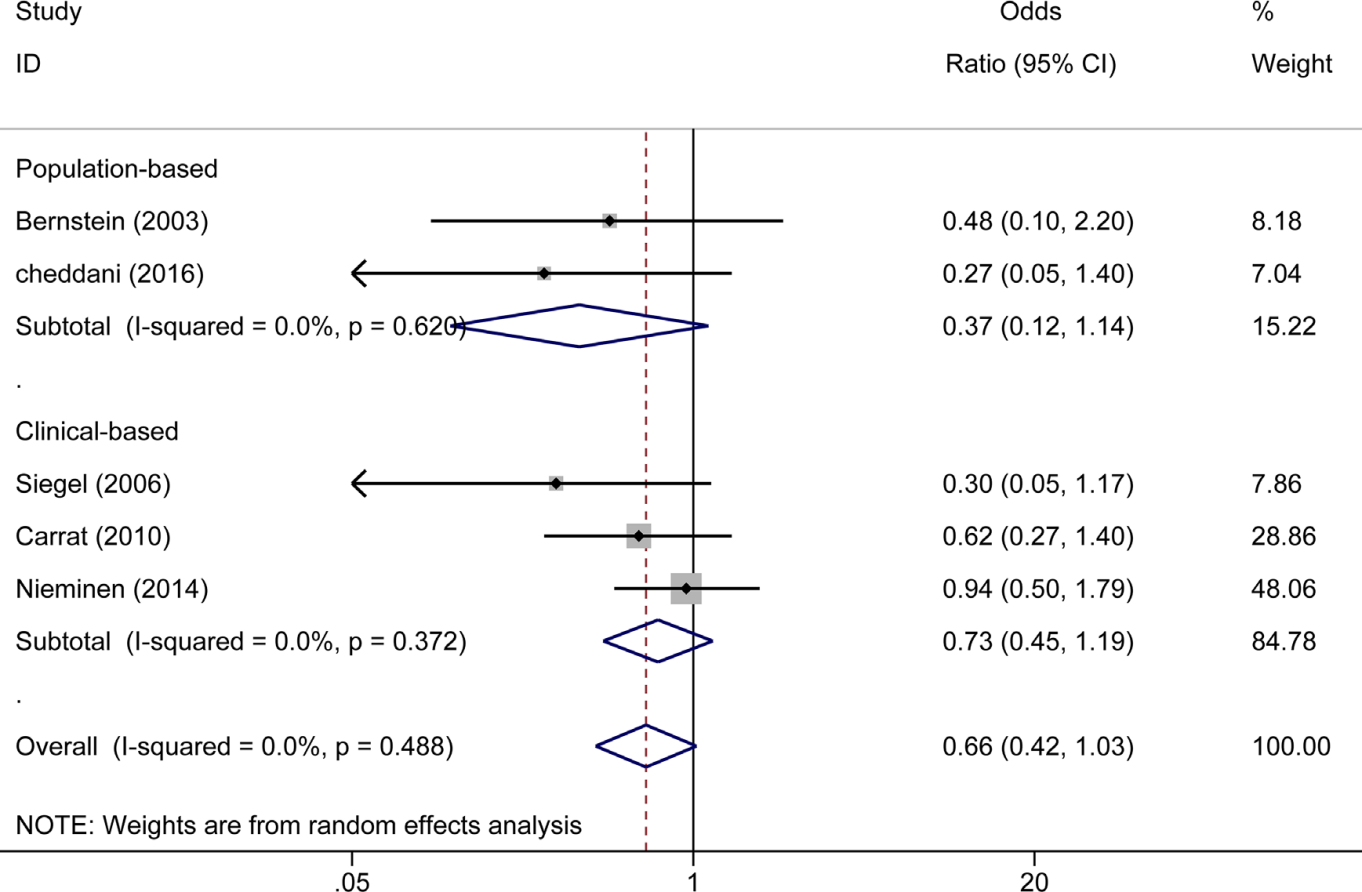
Forest plot of sub-analyses of 5-ASA on the risk of CRC/Dys in patients with CD between population-based and clinical-based cohort studies

**Figure 7:**
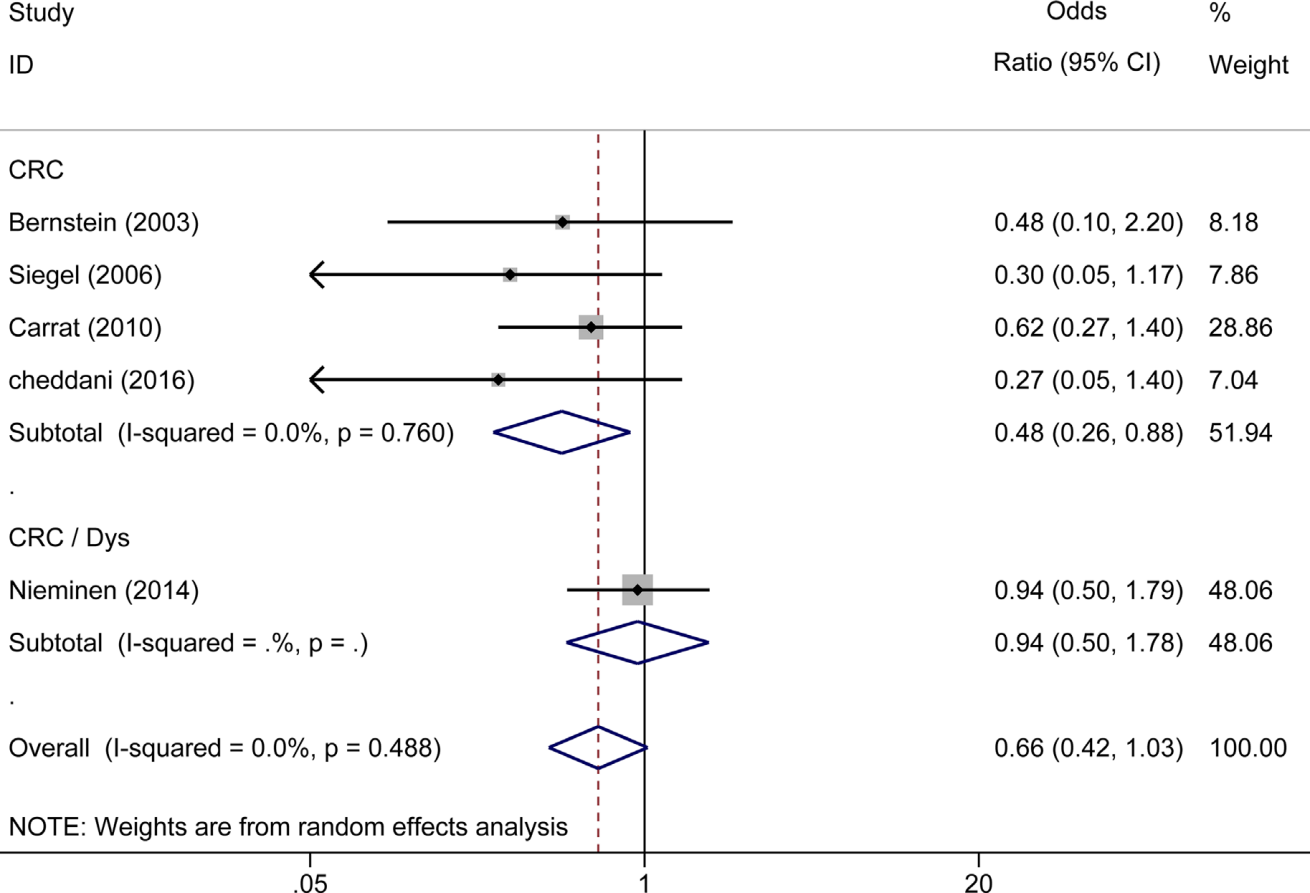
Forest plot of sub-analyses of 5-ASA on the risk of CRC, Dys and CRC/Dys in patients with CD

Five studies (two were population-based [22, 29] and three were clinical-based [11, 19, 34]) reported the effect of 5-ASA in patients with CD. Pooled analysis showed that 5-ASA had no protective effects in both the population-based (OR_population-based_ = 0.37, 95% CI: 0.12–1.14, *I^2^* = 0.0%, *P* = 0.620) and clinical-based (OR_clinical-based_ = 0.73, 95% CI: 0.45–1.19, *I^2^* = 0.0%, *P* = 0.372) studies. Pooling the five studies together yielded an OR of 0.66 (95% CI: 0.42–1.03, *I^2^* = 0.0%, *P* = 0.488) (Figure 6). Interestingly, four [11, 22, 29, 34] of the five studies used CRC as an outcome, revealing a reduced risk of CRC in the CD patients who received 5-ASA (OR_CRC_ = 0.48, 95% CI: 0.26–0.88, *I^2^* = 0.0%, *P* = 0.76). On the other hand, only one study [19] reported Dys and CRC as a combined outcome and showed that 5-ASA had no preventive effect on CRC/Dys in CD patients (OR_Dys_ = 0.94, 95% CI: 0.50–1.78) (Figure 7).

### Effect of different doses of 5-ASA on the risk of CRC/Dys in IBD patients

**Figure 8:**
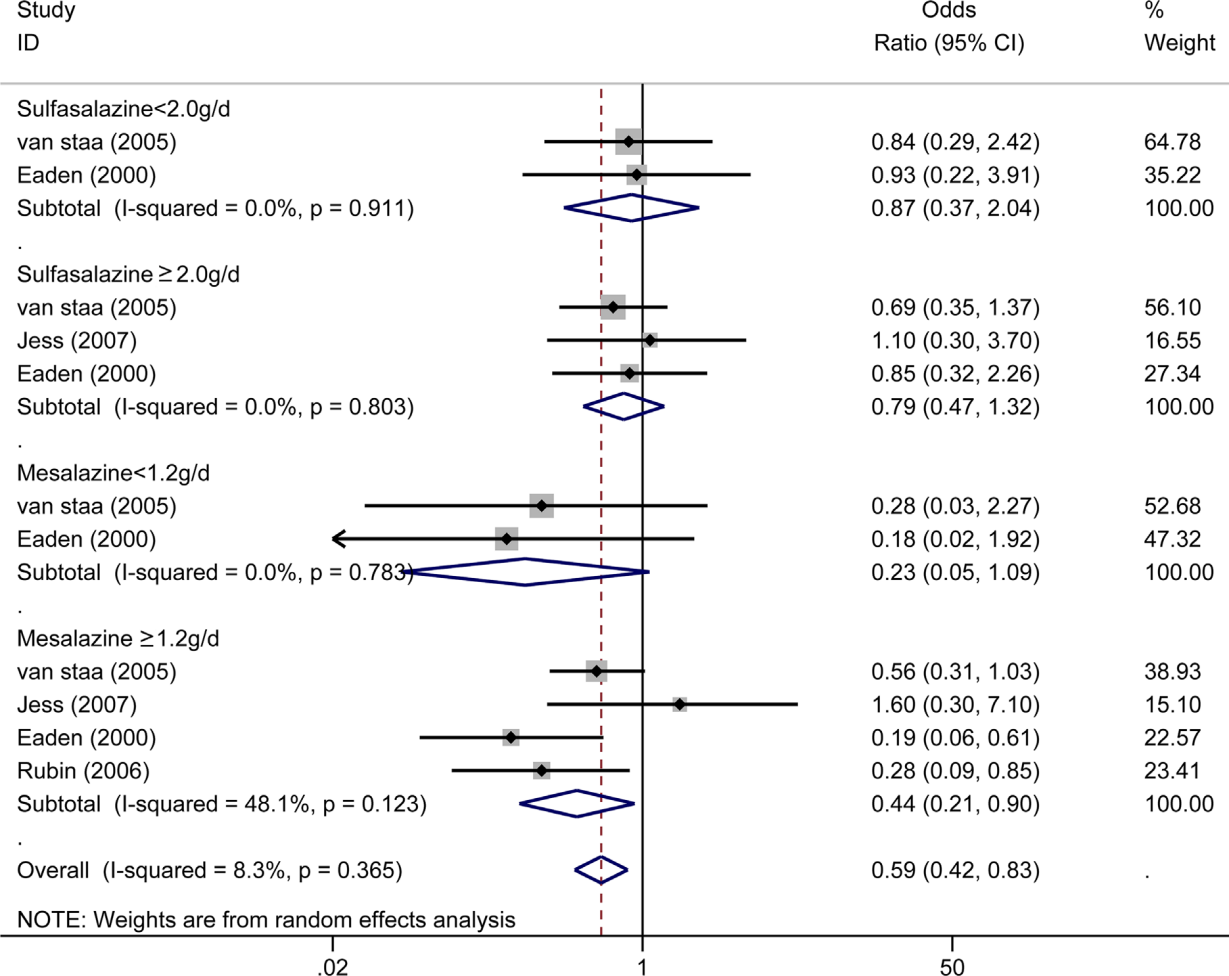
Forest plot of sub-analyses of 5-ASA (sulfasalazine and mesalazine) with different dosages (for sulfasalazine, daily dosage less than 2.0 g/d and above 2.0 g/d were estimated separately; for mesalazine, daily dosage less than 1.2 g/d and above 1.2 g/d were estimated separately) on the risk of CRC/Dys in patients with IBD

Three studies [9, 14, 32] reported the effect of sulphasalazine on the risk of CRC/Dys. Interestingly, sulfasalazine treatment showed no significant protective effect on reducing the risk of CRC/Dys in IBD patients treated with both high and low daily doses of sulphasalazine (≥ 2.0 g/day, OR = 0.79, 95%CI: 0.47–1.32, *I^2^* = 0.0%, *P* = 0.803; < 2.0 g/day, OR = 0.87, 95% CI: 0.37–2.04, *I^2^* = 0.0%, *P* = 0.911) in this meta-analyses. Four studies [9, 12, 14, 32] reported the effect of mesalazine on the risk of CRC/Dys. Patients received mesalamine ≥ 1.2 g/day had a lower risk (OR = 0.44, 95% CI: 0.21–0.90, *I^2^* = 48.1%, *P* = 0.123) of CRC/Dys compared with the patients received lower daily doses (< 1.2 g/day) of mesalazine (OR = 0.23, 95% CI: 0.05–1.09, *I^2^* = 0.0%, *P* = 0.783) (Figure 8).

### Effect of 5-ASA on the risk of CRC/Dys in IBD patients in different geographical regions

**Figure 9:**
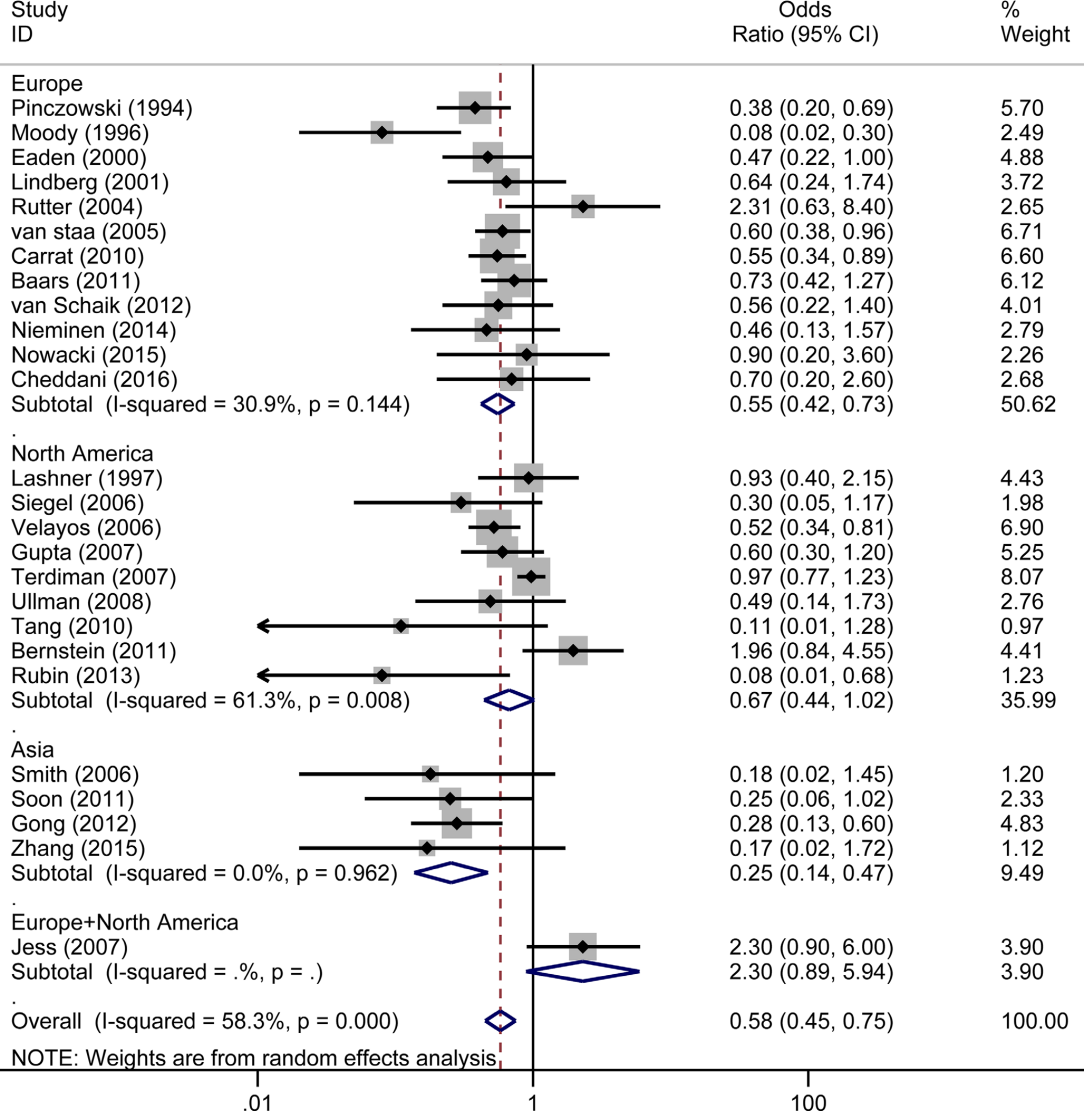
Forest plot of sub-analyses of 5-ASA on the risk of CRC/Dys in IBD patients from different geographical regions

Twelve [7–9, 11, 13, 16, 19, 20, 22, 32, 33, 41] of the 26 studies were conducted in Europe, nine [10, 12, 15, 29, 31, 34, 36–38] were conducted in North America, four [21, 35, 39, 40] were conducted in Asia, and one [14] was conducted in both Europe and North America. The chemo-preventive effect of 5-ASA was exhibited in the studies conducted in Asia (OR_Asia_ = 0.25, 95% CI: 0.14–0.47, *I^2^* = 0.0%, *P* = 0.962) and Europe (OR_Europe_ = 0.55, 95% CI: 0.42–0.73, *I^2^* = 30.9%, *P* = 0.144). However, no significant beneficial role of 5-ASA in preventing CRC/Dys was shown in studies conducted in North America (OR_North America_ = 0.67, 95% CI: 0.44–1.02, *I^2^* = 61.3%, *P* = 0.008). In addition, the study included mixed patients in Europe and North America was 2.30 (95%CI: 0.90–6.00). (Figure 9).

### Publication bias

Supplementary Figure 1 shows that publication bias existed in the 26 studies that evaluated the effect of 5-ASA in patients with IBD (A: Begg's test, *P* = 0.146; B: Egger's test, *P* = 0.023). However, when the effect of 5-ASA was studied only in UC patients, no significant bias was observed (A: Begg's test, *P* = 0.174; B: Egger's test, *P* = 0.251) (Supplementary Figure 2).

## DISCUSSION

This meta-analysis evaluates the effect of 5-ASA on CRC/Dys in patients with IBD. The pooled analysis of the 26 included studies showed that 5-ASA was associated with a decreased risk of CRC/Dys in patients with IBD in clinical-based studies, but not in population-based studies. This observation is in line with the conclusions reported by two previous meta-analyses [18, 24]. Several reasons could account for the differences observed between the clinical-based and population-based studies. First, the patients included in clinical-based studies usually had more severe disease activity and have a higher CRC/Dys risk than those included in population-based studies. Second, the inpatients usually take medications more regularly, and their confounding risk factors (e.g. extent of diseases, severity, comorbidities, and other medications) could be better recorded than those of patients in the communities [18]. Third, clinical-based studies are more susceptible to selection bias if the controls are not selected from the same source population as the cases. Consequently, the effect of 5-ASA on preventing malignant transformation in the inflamed colon is not so significant in population-based studies as compared to that in clinical-based studies. Notably, the heterogeneity among the population-based studies (*I^2^* = 73.8%, *P* = 0.000; Figure 2) was much greater than that among the clinical-based studies (*I^2^* = 13.4%, *P* = 0.297; Figure 2). Hence, the results from the population-based studies should be interpreted with caution.

Dys was the earliest histologically recognisable precursor of CRC [42]. Some clinical studies report both CRC and Dys as a combined end point (CRC/Dys), while the others still report CRC and Dys separately. In this meta-analysis, we performed a further sub-analysis for the different outcomes of CRC, Dys, and CRC/Dys in patients with IBD. Interestingly, in this sub-analysis, 5-ASA treatment had chemo-preventive effect only on CRC in IBD patients, but not on Dys or CRC/Dys. These novel findings could be explained as follows. First, unlike CRC, Dys (especially low-grade Dys) occurs more frequently in the flat colonic mucosa; however, no abnormalities detectable by conventional colonoscopy were present [43]. Hence, many dysplastic lesions may be missed. Second, patients usually have colitis during the diagnosis. Therefore, the dysplastic lesion may occur before a sufficient treatment course of 5-ASA, whereas CRC usually occurs in IBD patients with a disease duration > 8 years (and probably with a longer duration of 5-ASA use) [42]. Third, the subjects could develop sporadic adenomatous polyps and similar lesions that may not be related to IBD but could interfere with the investigations (on the effects of 5-ASA against Dys in IBD). In contrast, the incidence of sporadic CRC is much lower than that of Dys. Therefore, the interference while studying the chemopreventive effect of 5-ASA could be much lesser on CRC than on Dys.

Because UC and CD have quite different characteristics [44], we subsequently performed a sub-analysis on the risk of CRC/Dys development in UC and CD. Notably, 5-ASA treatment was associated with a reduced risk of CRC/Dys in UC patients in both population-based (*n* = 3) and clinical-based (*n* = 14) sub-analysis. (OR_population-based_ = 0.23, 95% CI: 0.09–0.60; OR_clinical-based_ = 0.52, 95% CI: 0.39–0.68). We also analyzed the risks of CRC and Dys in UC patients respectively. 5-ASA therapy significantly reduced the risk of CRC (OR = 0.40, 95% CI: 0.30–0.55) in UC patients, but cannot reduce the risk of Dys (OR = 0.18, 95% CI: 0.02–1.53) or combined CRC/Dys (OR = 0.62, 95% CI: 0.36–1.06) outcome. This study updated the meta-analysis published 10 years ago [23]. Since the larger sample size used in the present meta-analysis can decrease the random error risk in the interventions, the conclusions obtained are more reliable. Five published studies comprising 852 subjects analyzed the risk of CRC and Dys in patients with CD. In this meta-analysis, pooled analysis showed that 5-ASA did not reduce the risk of CRC/Dys in both population-based (*n* = 2) and clinical-based (*n* = 3) sub-analysis. (OR_population-based_ = 0.37, 95% CI: 0.12–1.14; OR_clinical-based_ = 0.73, 95% CI: 0.45–1.19). However, when the risks of CRC and Dys in CD patients were analyzed respectively, 5-ASA was beneficial for preventing CRC (but not Dys or combined CRC/Dys) outcome only. Owing to the limited sample size, the conclusions obtained should be interpreted with caution.

In this meta-analysis, we also analyzed the effects of different dosages of two kinds of 5-ASA (sulphasalazine and mesalazine) therapy in preventing CRC/Dys risk. Greater protective effects were achieved from treatment with mesalazine at dosages of ≥ 1.2 g/day than that with lower dosages, which is in line with a previous clinical research comparing the anti-neoplastic activities of 5-ASA at different dosages (≥ 1.2 g/day vs. < 1.2 g/day) directly [12]. However, treatment with sulphasalazine was not associated with a reduced risk of CRC/Dys development regardless of the dosage used (≥ 2.0 g/day or < 2.0 g/day). High incidence of adverse events (including headache, nausea, and fatigue) [45], intolerance, and discontinuation of sulphasalazine in patients [45] could explain the loss of the chemoprotective effect of sulphasalazine in IBD. In addition, intestinal inflammation and CRC development are strongly associated with dysbiosis of the intestinal microbial-environment [46–48]. Since 5-ASA derivatives can be metabolised by colonic microbiota, the mechanisms underlying the effect of 5-ASA on the intestinal microbiological equilibrium, and consequently on the CRC development are unknown.

As anti-neoplastic effects of 5-ASA could be varied across different geographical regions, we classified the studies mainly into three sub-groups according to the region where the studies were conducted (Europe, North America, and Asia). Notably, the best anti-neoplastic effect of 5-ASA use was found in patients from Asian countries, followed by the patients from European countries. However, 5-ASA did not show a significant protective effect in IBD patients from North America. The reason for this discrepancy is unknown. The differences in dietary habit, lifestyle, genetic susceptibility, and racial types may be involved in some of these inconsistencies.

The risk of neoplastic progression in IBD is multifactorial. Many studies included in this meta-analysis controlled several risk factors (gender, age, time since IBD diagnosis, disease severity, and extent of IBD) between the cases and controls, whereas a few of them controlled the family history of CRC, coexisting primary sclerosing cholangitis (PSC), and smoking history. Therefore, in this study, we analyzed three risk factors related to the induction of CRC. We found that a family history of CRC in first-degree relatives (but not any other relatives) doubled the risk of developing CRC in IBD patients (OR = 1.98, 95% CI: 1.06–3.72) based on three of the included studies [14, 16, 32] (Supplementary Figure 3). PSC is considered strongly associated with UC. [50]. The pooled result based on six [10, 12, 13, 20, 32, 36] of the included 26 studies exhibited more than two-fold higher risk of CRC development in UC patients coexisting PSC than in those without PSC (OR = 2.21, 95% CI: 1.13–4.33; Supplementary Figure 4). This indicates that PSC was an additional risk factor for the development of CRC in UC. However, none of the studies reported (or provided data for evaluating) the relationship between PSC and CD. It has been observed that smoking can protect against initial UC development and ameliorate the disease; however, it plays a opposite role in the development of CD [51]. From the pooled results based on six [7, 10, 12, 13, 31, 32] studies included, we found that UC patients with history of smoking showed a significantly reduced risk of CRC/Dys (OR = 0.71, 95% CI: 0.52–0.97; Supplementary Figure 5). However, one study [34] reported a detrimental effect of smoking in preventing CRC/Dys in patients with CD (OR = 2.15, 95% CI:0.72–6.47).

Several limitations should be considered in this meta-analysis. First, the *P* value from the Egger's test was lower than 0.05, implying that a publication bias could exist in the 26 studies conducted in patients with IBD. As papers with remarkable significance are usually likely to be published, unpublished studies (probably with negative results) were not included in this meta-analysis. However, no significant bias was found when the effect of 5-ASA was studied in patients with UC only. Second, all the studies included in this meta-analysis were observational studies. Furthermore, the studies differed in their design (case–control or cohort), study population (population- or clinical-based), outcomes evaluated (CRC, Dys, or CRC/Dys), year of publication, and the definition of exposure to 5-ASA (current use; former use; regular use; irregular use; use for > 3 months, > 1 year, > 5 years, > 10 years, > 20 years; use of different kinds of 5-ASA derivatives; and use of different 5-ASA dosages). Therefore, there were significant heterogeneities among the studies, and it was difficult to divide them into reasonably sized groups for stratified analysis based on all the exposure definitions. Third, many of the confounding factors (e.g. disease course/severity/location and concomitant medication) were not controlled in some of the studies included, and the sample size of several subgroups was small. In addition, it remains unclear whether treatment with other anti-inflammatory drugs (e.g. glucocorticoids, immunomodulators, and biologics) could improve CRC prevention. Moreover, the risk factors for CRC/Dys in patients with IBD should be considered during the treatment of this disease.

In summary, our pooled analysis demonstrated that 5-ASA has a chemo-preventive effect against CRC (but not Dys) in patients with IBD. Moreover, patients with UC can benefit more from 5-ASA therapy than patients with CD. Mesalazine maintenance dosages ≥ 1.2 g/day is an effective treatment for reducing cancer risk in IBD patients, while larger cohorts of IBD patients over a longer follow-up period (probably more than 20 years) are warranted to make this conclusion more reliable. Therefore, early diagnosis, long-term adherence to colonoscopic surveillance, and prompt anti-inflammatory therapy should be initiated in these IBD patients (especially those with high risk of CRC), to block CRC at an earlier stage.

## MATERIALS AND METHODS

### Search strategies

We searched Web of Science, PubMed, Cochrane CENTRAL, Medline (Ovid), and Embase for all relevant articles and abstracts, published up to August 2016, regarding the effect of 5-ASA treatment on the risk of developing CRC or dysplasia among IBD patients. The applied English Medical Subject Headings (MeSH) or free text terms used in the research included ‘ulcerative colitis’, ‘Crohn's disease’, ‘inflammatory bowel disease’, ‘5-ASA’, ‘5-aminosalicylate’, ‘5-aminosalicylic acid’, ‘mesalamine’, ‘mesalazine’, ‘sulfasalazine’, ‘sulphasalazine’, ‘olsalazine’, ‘balsalazide’, ‘colonic cancer’, ‘colorectal cancer’, ‘dysplasia’, and ‘neoplasia’.

### Study selection

Two authors (XQ and JM) independently selected relevant clinical trials following the proposal for reporting Meta-analyses of Observational Studies in Epidemiology (MOOSE) guidelines [52]. They then discussed with the senior author (HZ) whether the studies should be included in the analysis. Studies were included if they: reported on subjects diagnosed with IBD with colonic involvement (either UC or Crohn's disease); evaluated exposure to 5-ASA in IBD patients; reported CRC or Dys outcomes; reported odds ratios (ORs), relative risks (RRs), or hazard ratios (HRs) with 95% confidence intervals (CI) or provided the relevant data for calculating those effect measures; or were case–control or cohort studies. Inclusion was not otherwise restricted by language, study size, or publication type. If a study group published more than one article using the same case series, data from the most recent comprehensive report were included in the analysis unless that the data could not be retrieved from it.

### Data extraction and quality assessment

The following data were extracted from each study: the first author's name, year of publication, study period, numbers of cases and controls, total numbers of cases in each group, study design (case–control or cohort), distribution of IBD diagnosis (UC and CD), categories of 5-ASA, outcomes (CRC/Dys), study settings (population-based or clinical-based), and country of origin. The ORs, RRs, or HRs with and without adjustment for potential confounders and the corresponding 95% CIs in each study were separately recorded, either directly from the included studies or by contacting the original author by email. Disagreements were resolved by consensus with a third author (HZ). The Newcastle-Ottawa Scale (NOS) was used to evaluate the quality of each study [53]. An ultimate score of ≥ 6 stars was regarded as high quality.

### Statistical analysis

Adjusted data for gender, age, time since IBD diagnosis, and severity and extent of IBD in the included studies were prior adopted, unless only unadjusted (crude) data were provided. Because the incidence of CRC/Dys in patients with IBD was relatively low, we considered the OR value to be mathematically approximate to the RR and HR values. When the estimates (including OR, RR, or HR) of different types of 5-ASA (sulphasalazine and non-sulphasalazine) were separately reported in the same study, the overall estimate of 5-ASA was pooled based on the individual estimate for each subgroup. The association between 5-ASA and IBD-associated CRC/Dys was quantified by using the DerSimonian and Laird random-effects model.

The *Q* and *I^2^* statistics were used to test statistical heterogeneity among the studies. For the *Q* statistic, heterogeneity was considered as statistically significant if a *P* value assessed by using chi-square test was less than 0.10. In addition, the analysis yielded *I^2^* indices that ranged from 0% to 100%. Heterogeneity was considered very low if the *I*^2^ index was about 0–25%, low if about 25–50%, medium if about 50–75%, and high if about 75–100%. Subgroup analyses based on study settings (population-based or clinical-based), disease type (UC, CD, or UC/CD), and 5-ASA (sulphasalazine and mesalazine) dosage were evaluated. Begg's funnel plot and Egger's test were performed to evaluate publication bias. Data were analysed using Stata software version 12.0 (StataCorp, College Station, TX, USA).

## SUPPLEMENTARY MATERIALS


