# Supplementary material for: Chemopreventive effects of 5-aminosalicylic acid on inflammatory bowel disease-associated colorectal cancer and dysplasia: a systematic review with meta-analysis
Source: Oncotarget. 2016 Nov 30;8(1):1031–45. doi: 10.18632/oncotarget.13715 (PMC5352032; doi:10.18632/oncotarget.13715)
Supplement: Supplementary file 1 [file oncotarget-08-1031-s001.pdf]

Chemopreventive effects of 5-aminosalicylic acid on inflammatory bowel disease-associated colorectal cancer and dysplasia: a systematic review with meta-analysis

Supplementary Materials

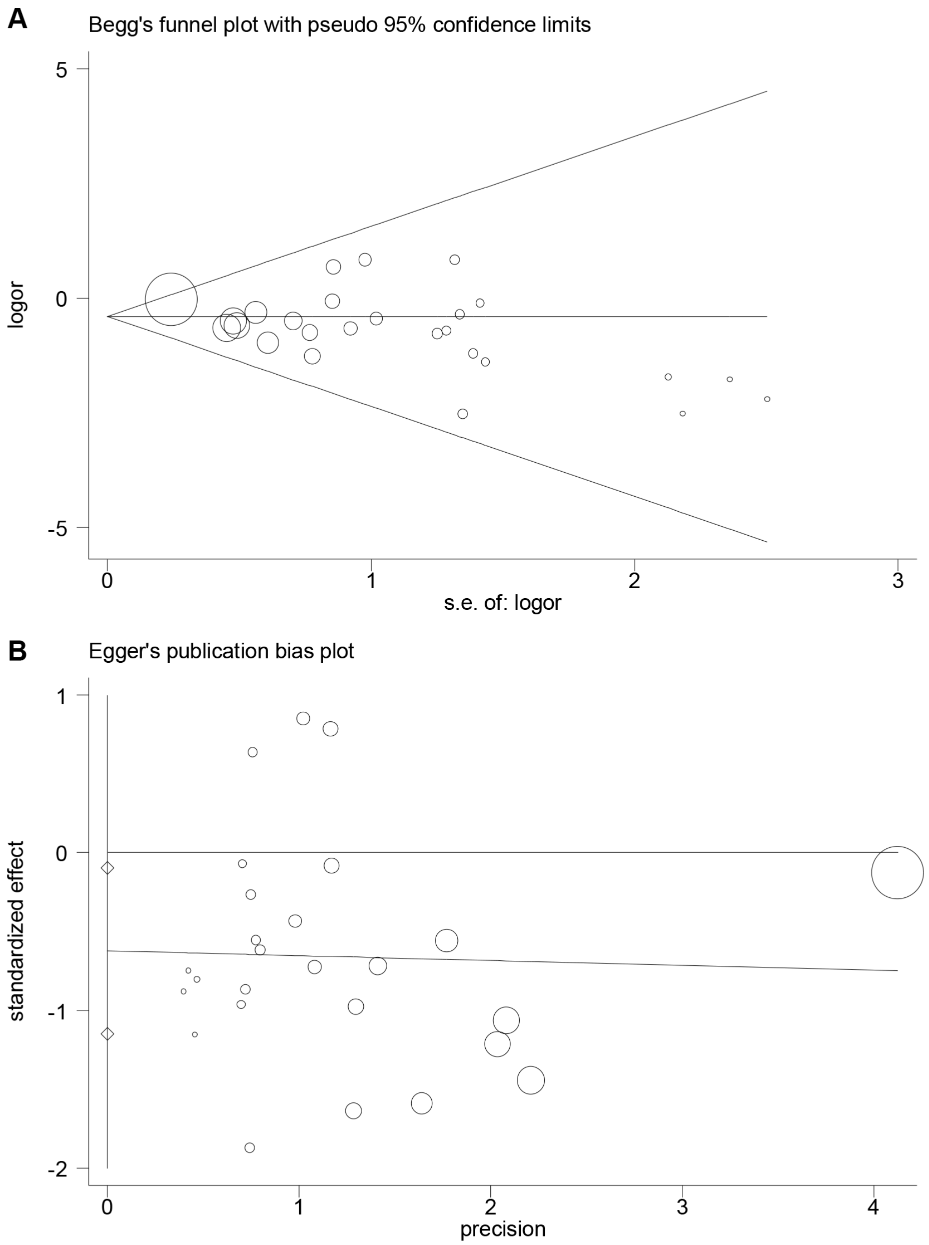

Supplementary Figure S1: Begg's test (**A**) identified no significant publication bias ($p = 0.146$) while Egger's test (**B**) showed significant publication bias ($p = 0.023$) in the total 26 studies on patients with IBD (UC/CD)**.**

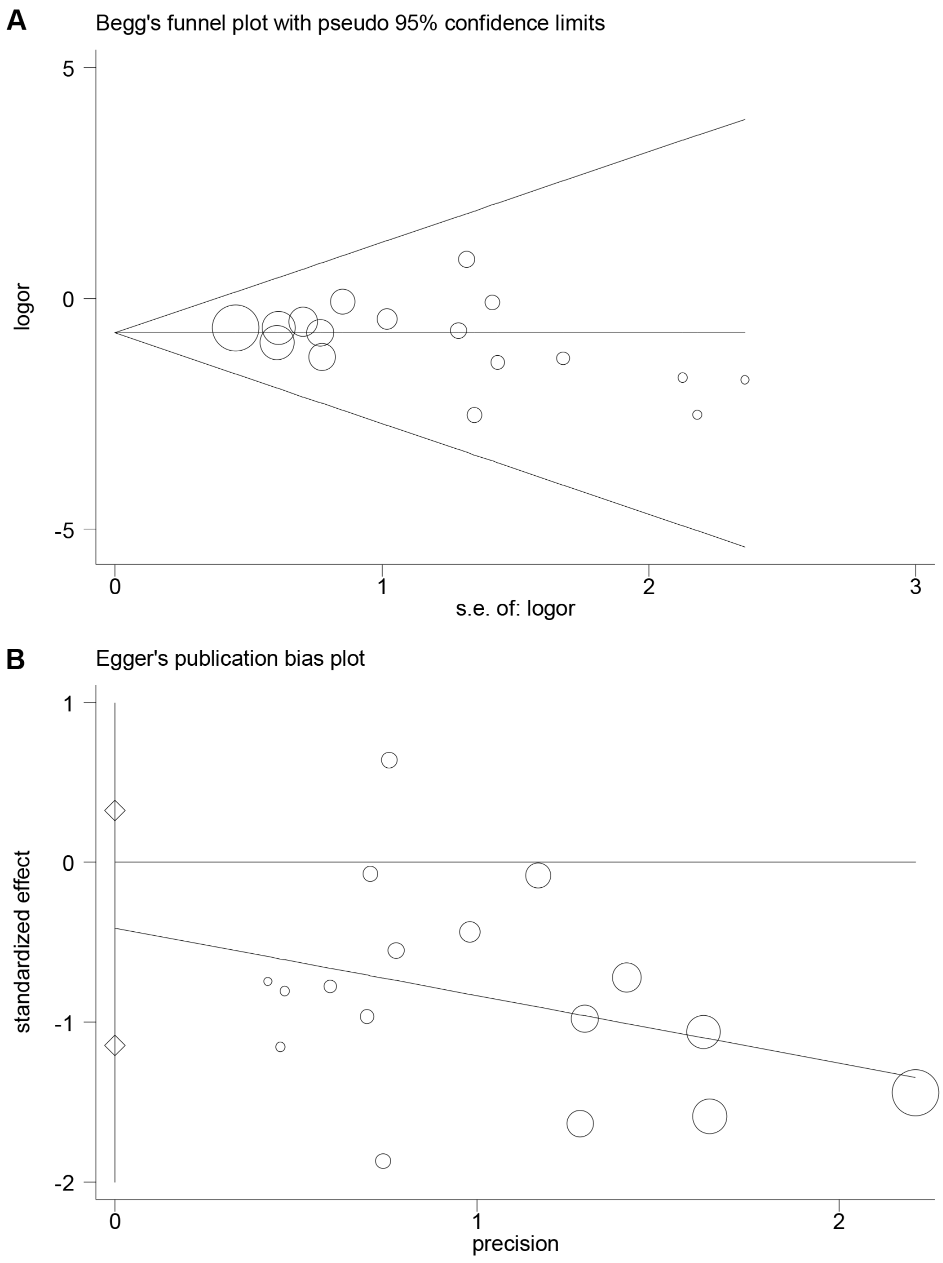

Supplementary Figure S2: Both Begg's test (**A**) and Egger's test (**B**) showed no significant publication bias (p = 0.174 and 0.251, respectively) in the total 17 studies on patients with UC**.**

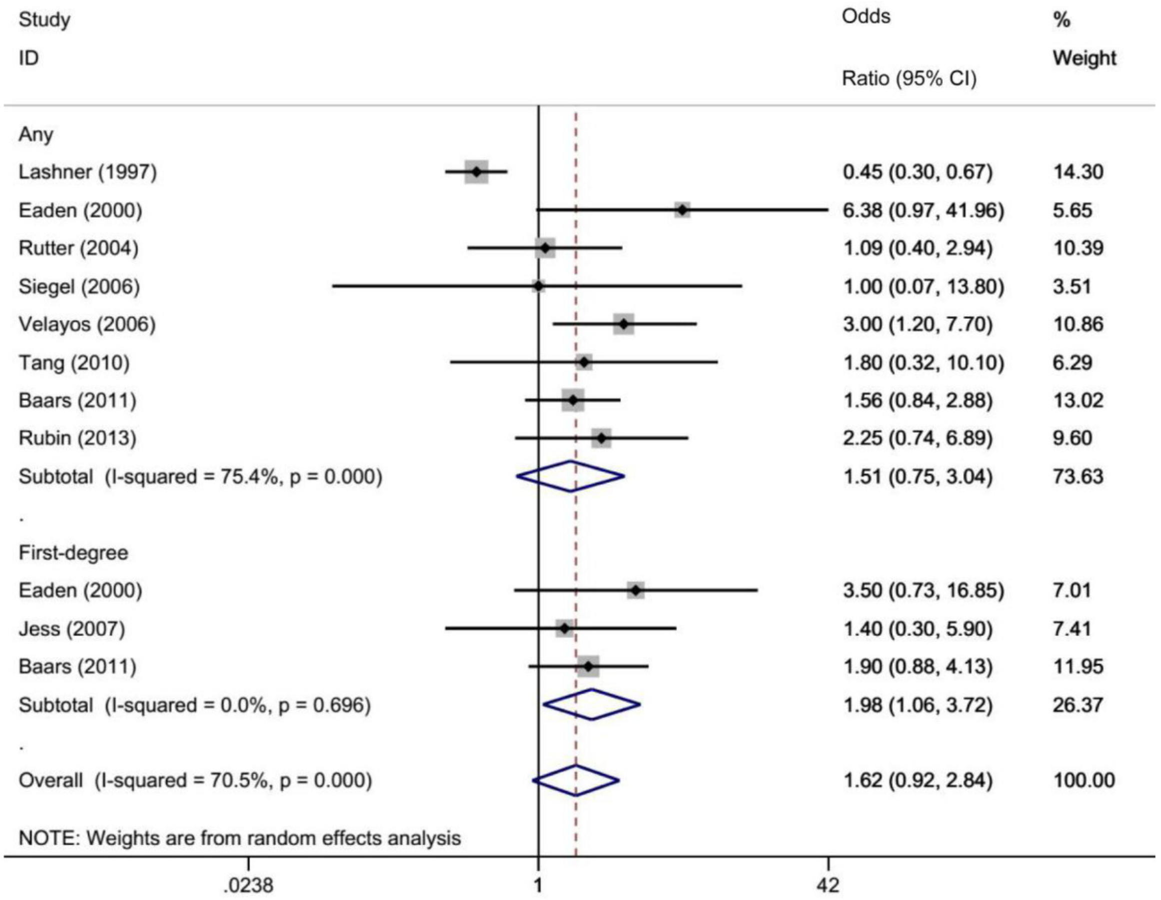

Supplementary Figure S3: Risk of developing CRC/Dys in IBD patients with family history of CRC in any or first-degree relatives.

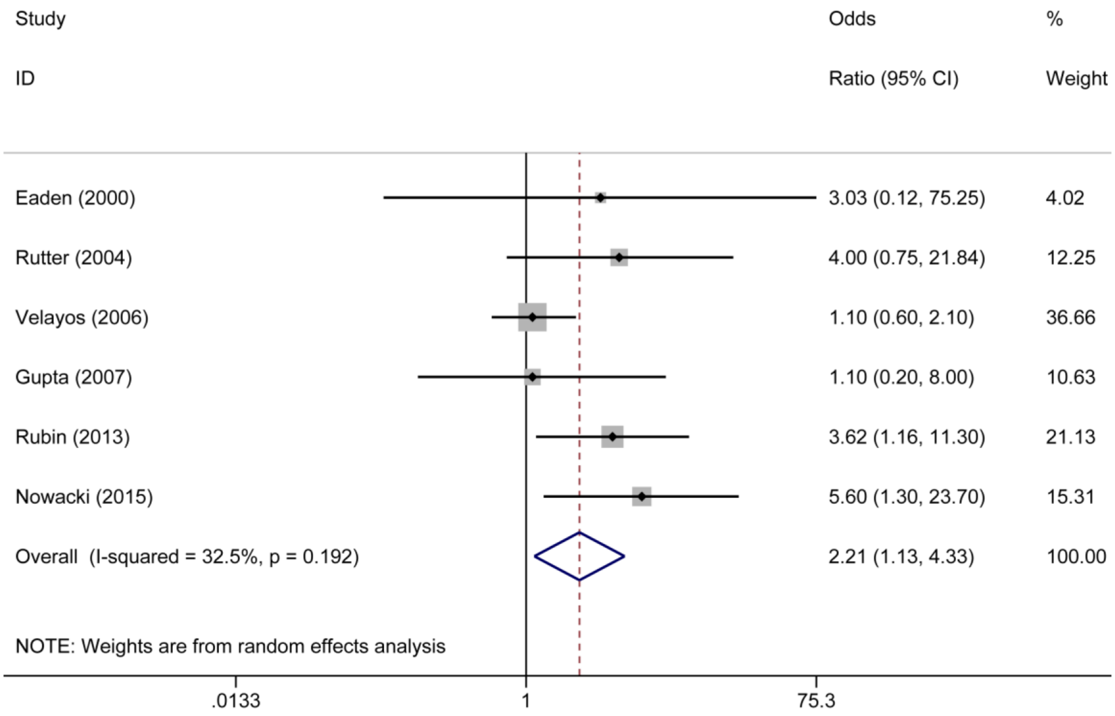

Supplementary Figure S4: Risk of developing CRC/Dys in UC patients with coexisting primary sclerosing cholangitis (PSC).

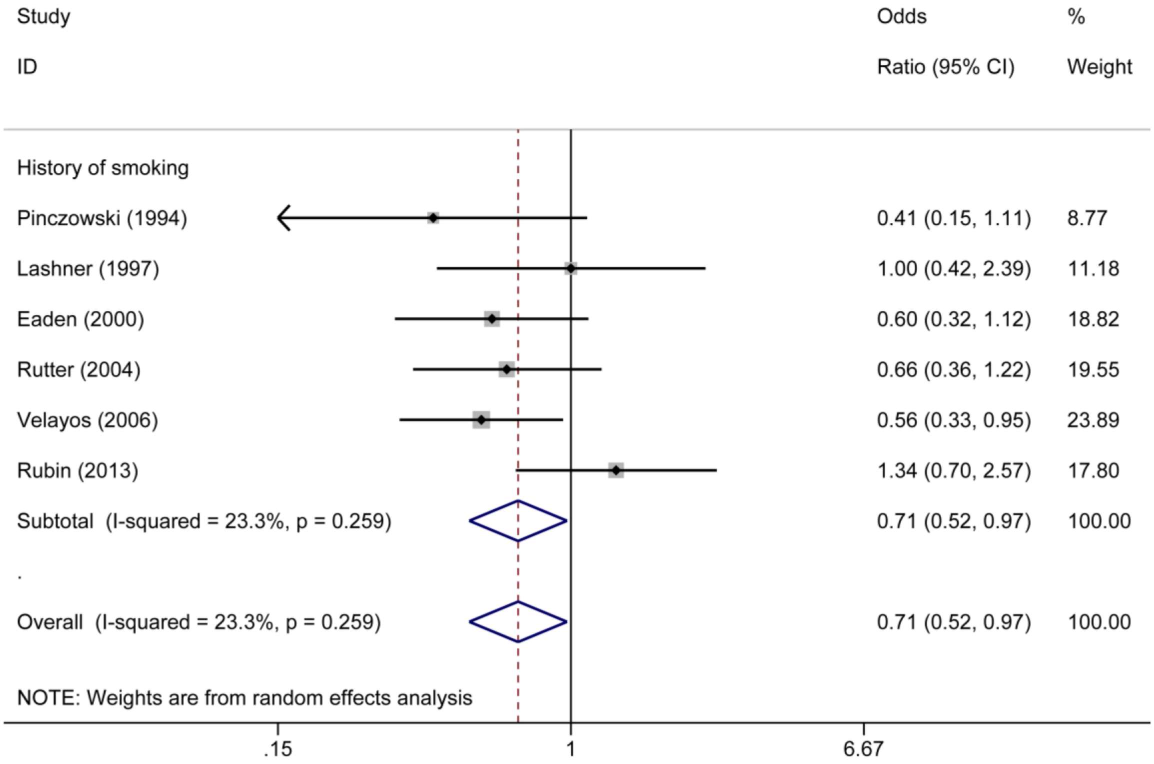

Supplementary Figure S5: Risk of developing CRC/Dys in UC patients with smoking history.
